# Body composition parameters in initial CT imaging of mechanically ventilated trauma patients: Single‐centre observational study

**DOI:** 10.1002/jcsm.13578

**Published:** 2024-08-26

**Authors:** Hans‐Jonas Meyer, Tihomir Dermendzhiev, Michael Hetz, Georg Osterhoff, Christian Kleber, Timm Denecke, Jeanette Henkelmann, Robert Werdehausen, Gunther Hempel, Manuel F. Struck

**Affiliations:** ^1^ Department of Diagnostic and Interventional Radiology University Hospital Leipzig Leipzig Germany; ^2^ Department of Orthopedics, Trauma and Plastic Surgery University Hospital Leipzig Leipzig Germany; ^3^ Department of Anesthesiology and Intensive Care, Medical Faculty University of Magdeburg Magdeburg Germany; ^4^ Department of Anesthesiology and Intensive Care Medicine University Hospital Leipzig Leipzig Germany

**Keywords:** Polytrauma, Emergency diagnostics, Whole‐body CT, Body composition parameters, Mortality

## Abstract

**Background:**

Body composition parameters provide relevant prognostic significance in critical care cohorts and cancer populations. Published results regarding polytrauma patients are inconclusive to date. The goal of this study was to analyse the role of body composition parameters in severely injured trauma patients.

**Methods:**

All consecutive patients requiring emergency tracheal intubation and mechanical ventilation before initial computed tomography (CT) at a level‐1 trauma centre over a 12‐year period (2008–2019) were reanalysed. The analysis included CT‐derived body composition parameters based upon whole‐body trauma CT as prognostic variables for 30‐day mortality, intensive care unit length of stay (ICU LOS) and mechanical ventilation duration.

**Results:**

Four hundred seventy‐two patients (75% male) with a median age of 49 years, median injury severity score of 26 and 30‐day mortality rate of 22% (104 patients) met the inclusion criteria and were analysed. Regarding body composition parameters, 231 patients (49%) had visceral obesity, 75 patients had sarcopenia (16%) and 35 patients had sarcopenic obesity (7.4%). After adjustment for statistically significant univariable predictors age, body mass index, sarcopenic obesity, visceral obesity, American Society of Anesthesiologists classification ≥3, injury severity score and Glasgow Coma Scale ≤ 8 points, the Cox proportional hazard model identified sarcopenia as significant prognostic factor of 30‐day mortality (hazard ratio 2.84; 95% confidence interval 1.38–5.85; *P* = 0.004), which was confirmed in Kaplan–Meier survival analysis (log‐rank *P* = 0.006). In a subanalysis of 363 survivors, linear multivariable regression analysis revealed no significant associations of body composition parameters with ICU LOS and duration of mechanical ventilation.

**Conclusions:**

In a multivariable analysis of mechanically ventilated trauma patients, CT‐defined sarcopenia was significantly associated with 30‐day mortality whereas no associations of body composition parameters with ICU LOS and duration of mechanical ventilation were observed.

## Introduction

The life‐threatening conditions of severely injured trauma patients require rapid and comprehensive clinical diagnosis to initiate the earliest possible treatment and risk stratification.^1^ In the last three decades, whole‐body computed tomography (CT) has been established as the clinical gold standard of emergency diagnostics and is performed particularly in polytrauma patients suspected of having multiple injuries and in complex injury patterns.^2^, ^3^, ^4^ Although uncritical use of CT may lead to possibly harmful radiation exposure, there is consensus that severely injured trauma patients benefit from early CT imaging.^5^, ^6^, ^7^ Due to the reliable detection of acute bleeding events, brain injuries, solid organ injuries, pneumothorax, spine injuries, pelvis fractures and long bone fractures, CT findings serve as the main source for calculating established scoring systems, including the abbreviated injury scale (AIS) and injury severity score (ISS).^1^

In addition to the anatomical factors detected by diagnostic imaging, prognosis prediction and guidance of therapy depend on assessment of physiological risk factors, including age, pre‐injury condition [American Society of Anesthesiologists (ASA) classification], and level of consciousness [Glasgow Coma Scale (GCS)].^8^, ^9^ Furthermore, the need for emergency tracheal intubation after injury is a treatment‐related indicator of injury severity.^10^, ^11^, ^12^, ^13^

Analysis of body composition parameters, byproducts derived from CT diagnostics, is an emerging research field in which different morphological structures, particularly fat and skeletal muscle tissue mass areas, are adjusted to body height and sex.^14^ These clinical surrogate parameters of visceral obesity, sarcopenia and sarcopenic obesity provide prognostic significance, particularly for cancer populations but also in various emergency conditions.^15^, ^16^ Their application and possible benefit in trauma patients have not been explored sufficiently, and results are inconclusive.^17^, ^18^, ^19^

The purpose of the present study was to analyse associations of body composition with 30‐day mortality and intensive care unit (ICU) treatment in a homogeneous cohort of patients with severe injuries requiring emergency tracheal intubation and mechanical ventilation. We hypothesized that CT‐derived body composition data would be prognostically relevant in severely injured patients and therefore add to the already known diagnostic value of initial emergency CT for injury assessment.

## Materials and methods

### Patient enrolment

After approval by the Ethics Committee of the Medical Faculty of the University of Leipzig, consecutive trauma patients of the University Hospital Leipzig were retrospectively analysed with special regard to body composition parameters and possible associations with outcomes. The inclusion criteria were (1) direct admission from the scene to the emergency department (ED) by emergency medical services (EMS) between 2008 and 2019; (2) tracheal intubation and mechanical ventilation in the pre‐hospital setting or in the trauma room; (3) initial whole‐body CT diagnostics performed; (4) admission to the ICU, including patients with and without emergency surgery immediately after CT; and (5) availability of demographic characteristics, ISS and GCS scores. The exclusion criteria were incomplete or missing data, patients <18 years of age and CT imaging without the use of contrast media.

### Investigated parameters

Demographic parameters included age, impaired pre‐injury condition (ASA classification ≥III) and body mass index (BMI). Injury severity was classified using the ISS (derived from AIS of body regions head, face, chest, abdomen, extremities and external region) and impaired consciousness before tracheal intubation (GCS ≤ 8 points). All variables were calculated by the attending teams and documented in the electronic patients charts. ICU length of stay (LOS), duration of mechanical ventilation and all‐cause 24‐h and 30‐day mortality were assessed.

### Imaging technique

Contrast‐enhanced CT was performed in a clinical setting using a 128‐slice CT scanner (Ingenuity 128, Philips, Hamburg, Germany). Iodine‐based contrast medium (90 mL Imeron® 400 MCT, Bracco Imaging Germany GmbH, Konstanz, Germany) was administered intravenously at a rate of 2–4.0 mL/s. Automatic bolus tracking was performed in the descending aorta with a trigger of 100 Hounsfield units (HU). CT images were obtained in the late arterial phase in every case. Typical imaging parameters were as follows: 100 kVp; 125 mAs; slice thickness, 1 mm; and pitch, 0.9. The CT covered the head to the upper thighs.

### Body composition assessment

Body composition parameters were measured with ImageJ software 1.48v (National Institutes of Health Image program). Every parameter was measured at the lumbar 3 (L3) level. The skeletal muscle area (SMA) included the psoas muscle, paraspinal muscles and abdominal wall muscles. SMA was semi‐automatically measured using HU threshold levels of −29 and 150 HU, as defined previously.^20^ The SMA was divided by height squared to calculate the skeletal muscle index (SMI). For sarcopenia definition, the SMI threshold defined by Prado et al. was used (52.4 cm^2^/m^2^ for males and 38.5 cm^2^/m^2^ for females).^16^

Visceral adipose tissue (VAT) and subcutaneous adipose tissue (SAT) were calculated at the same L3 level as the muscle areas. The fat areas were semi‐automatically measured using HU threshold levels of −190 and −30 HU.^21^ The VAT index (VATI) threshold defined by Baggerman et al. (>38.7 cm^2^/m^2^ for males and >24.9 cm^2^/m^2^ for females) was used to determine visceral obesity.^22^ Finally, sarcopenic obesity was defined for patients with sarcopenia and visceral obesity. Further exploratory threshold values were calculated for sarcopenia and visceral obesity using the best matching quartile of the study cohort associated with 30‐day mortality.

All measurements were performed by the second author under guidance of the first author.

Interreader agreement measurements were conducted using a randomly selected sample of 30 patients to ensure the reliability of the measurements. All 30 patients were measured by both authors independently from one another.

The results of other CT findings of the same study cohort, namely, coronary artery calcification and epicardial adipose tissue have been evaluated and published recently.^23^, ^24^

### Statistical analysis

The data analysis included testing for normality distribution using the Kolmogorov–Smirnov test. Categorical data were presented as numbers (percentages) while all metric data were presented using medians and interquartile ranges (IQR, quartile 1 and quartile 3) as the majority of the data were non‐normally distributed. Group comparisons for metric data were conducted using the Mann–Whitney *U* test or Student's *t* test, depending on the distribution. Group comparisons of categorical data were performed using the *χ* ^2^ test. Interreader agreement was calculated using the intra‐class correlation coefficient (ICC). A Cox proportional hazard model was applied to identify predictors of 30day mortality using selected statistically significant associations of univariable analyses. The number of investigated variables was estimated to provide at least ten events per variable (i.e., deaths). A Kaplan–Meier survival plot was calculated from ED admission to the time of mortality events and compared using the log‐rank test. To identify possible associations of body composition parameters with ICU LOS and duration of mechanical ventilation in survivors, multivariable linear regression analyses were performed, which included statistically significant predictors of univariable analyses. Cohort‐specific definitions of sarcopenia, visceral obesity and sarcopenic obesity were determined using the most significant quartiles of SMI and VATI adjusted to male and female patients' threshold values associated with 30‐day mortality. These definitions were applied in the same manner as the previously used definitions, using univariable and multivariable Cox regression analysis for associations with 30‐day mortality and univariable and multivariable linear regression analysis for ICU LOS and mechanical ventilation duration.

In all instances, *P* values <0.05 were considered statistically significant. Correction for type 1 error was applied using the false discovery rate (FDR). The statistical analysis was performed using DATAtab (DATAtab e.U., Graz, Austria), R 4.2.2 (R Foundation for Statistical Computing, Vienna, Austria) and GraphPad Prism version 10.0.2 (GraphPad Software, Boston, MA, USA).

## Results

**Figure 1:**
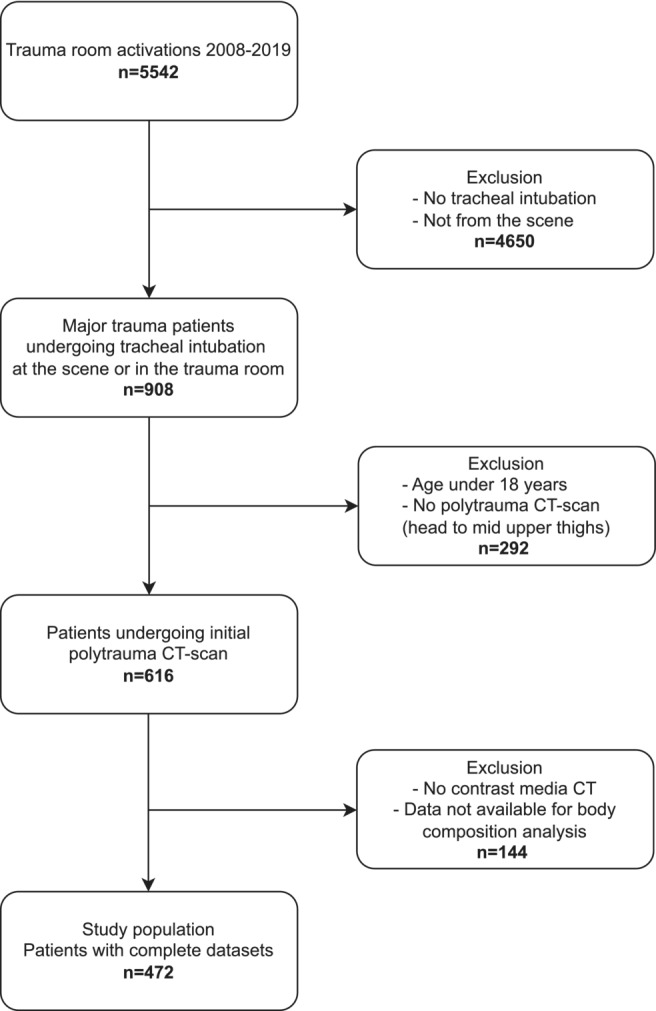
Study flow chart of the study. Overall, 472 patients were included into the present study.

Four hundred seventy‐two patients (354 male patients, 75%) with a median age of 49 years (20% of whom were aged ≥70 years) met the inclusion criteria and were analysed (*Figure* *1*). In the majority of patients (58%), traffic accidents were the cause of injury; 31% had falls from height, 8% had other blunt injuries and 3% had penetrating injuries. The median ISS was 26, and the proportion of patients with impaired consciousness (GCS ≤ 8 points) before tracheal intubation was 64.4%. All‐cause 24‐h and 30‐day mortality were 7.6% and 22%, respectively (*Table* *1*).

**Table 1:** Characteristics of the study cohort including comparison of survivors and non‐survivors

Parameter	All patients (*n* = 472)	Survivors (*n* = 368)	Non‐survivors (*n* = 104)	*P* value	FDR
Male, *n* (%)	354 (75)	276 (75)	78 (75)	1	1
Age, years; median (IQR)	49 (31; 63.25)	45 (30; 60.25)	59 (35; 75)	<0.001	<0.001
ASA ≥ III; *n* (%)	102 (21.6)	62 (16.5)	40 (38.5)	<0.001	<0.001
BMI, kg/m^2^; median (IQR)	25.2 (23.4; 27.7)	25.15 (23.1; 27.5)	25.7 (24.2; 27.8)	0.082	0.118
Sarcopenia; *n* (%)	75 (15.9)	50 (13.6)	26 (25)	0.01	0.02
Sarcopenia Q1; *n* (%)	115 (24.4)	80 (21.7)	35 (33.6)	0.012	0.022
Visceral obesity; *n* (%)	213 (45.1)	150 (40.7)	63 (60.6)	<0.001	<0.001
Visceral obesity Q2; *n* (%)	236 (50)	171 (46.5)	65 (62.5)	0.004	0.009
Sarcopenic obesity; *n* (%)	28 (5.9)	15 (4.1)	13 (12.5)	0.001	0.003
Sarcopenic obesity Q1_Q2; *n* (%)	51 (10.8)	29 (7.9)	22 (21.1)	<0.001	<0.001
SMM, cm^2^; median (IQR)	180.1 (153.45; 207.15)	182.3 (156.48; 206.83)	173.15 (142.1; 210.73)	0.102	0.139
SMI, cm^2^/m^2^; median (IQR)	57.55 (50.68; 64.4)	57.75 (51.5; 64.33)	57.1 (48.03; 65.03)	0.11	0.143
TAT, cm^2^; median (IQR)	238.25 (143.03; 360.1)	227.2 (141.13; 363.78)	272.6 (165.53; 354.1)	0.157	0.194
SAT, cm^2^; median (IQR)	127.3 (83.15; 181.5)	127.1 (86.1; 184.55)	128.35 (78.38; 169.95)	0.951	0.989
VAT, cm^2^; median (IQR)	97.65 (42.95; 179.43)	86.65 (38.98; 168.48)	136.7 (56.63; 193.9)	0.005	0.011
VATI, cm^2^/m^2^; median (IQR)	32.04 (13.76; 57.77)	28.23 (12.54; 52.71)	42.9 (18.07; 64.54)	0.003	0.008
ISS; median (IQR)	26 (20; 41)	25 (18; 34)	45 (34; 57.25)	<0.001	<0.001
AIS head; median (IQR)	3 (0; 5)	2 (0; 4)	5 (5; 5)	<0.001	<0.001
AIS face; median (IQR)	0 (0; 1)	0 (0; 1)	0 (0; 2)	0.541	0.639
AIS chest; median (IQR)	3 (0; 4)	3 (0; 4)	3 (0; 4)	0.023	0.037
AIS abdomen, lumbar spine; median (IQR)	0 (0; 2)	0 (0; 2)	0 (0; 3)	0.077	0.117
AIS extremity, pelvis; median (IQR)	2 (0; 3)	2 (0; 3)	1.5 (0; 3)	0.85	0.921
AIS external; median (IQR)	0 (0; 0)	0 (0; 0)	0 (0; 0)	0.761	0.86
GCS ≤ 8 points; *n* (%)	304 (64.4)	203 (55.1)	101 (97.1)	<0.001	<0.001
ICU LOS, days; median (IQR)	8 (3; 22)	11.5 (4; 27)	3 (1; 7)	<0.001	<0.001
Mechanical ventilation, days; median (IQR)	3 (0.5; 14)	4 (0.5; 16)	3 (0.94; 7)	0.016	0.028
24h mortality; *n* (%)	36 (7.6)		36 (34.6)		
30‐day mortality; *n* (%)	104 (22)		104 (100)		

*Note*: Sarcopenia defined by Prado et al.^14^; visceral obesity defined by Baggerman et al.^22^; Sarcopenic obesity defined using definitions of sarcopenia and visceral obesity.^14^, ^22^ Sarcopenia Q1, first quartile of sex‐adjusted SMI of the study cohort associated with 30‐day mortality; visceral obesity Q2, second quartile of sex‐adjusted VATI of the study cohort associated with 30‐day mortality; sarcopenic obesity Q1_Q2, sarcopenic obesity defined by cohort specific sarcopenia and visceral obesity definitions.

Abbreviations: AIS, abbreviated injury scale of body region; ASA, American Society of Anesthesiologists classification; BMI, body mass index; FDR, false discovery rate; GCS, Glasgow Coma Scale; ICU LOS, intensive care unit length of stay; IQR, interquartile range; ISS, injury severity score; SAT, subcutaneous adipose tissue; SMI, skeletal muscle index; SMM, skeletal muscle mass; TAT, total adipose tissue; VAT, visceral adipose tissue; VATI, visceral adipose tissue index.

According to the previously defined body composition definitions,^14^, ^22^ visceral obesity was present in 231 patients (49%), sarcopenia in 75 patients (16%) and sarcopenic obesity in 35 patients (7.4%).

All CT body composition measurements demonstrated an excellent interreader agreement with ICC values for SMI, SAT, VAT and TAT of 0.97 [95% confidence interval (CI) 0.94–0.98], 0.99 (95% CI 0.98–0.99), 0.98 (95% CI 0.97–0.99) and 0.99 (95% CI 0.97; 0.99), respectively.

**Figure 2:**
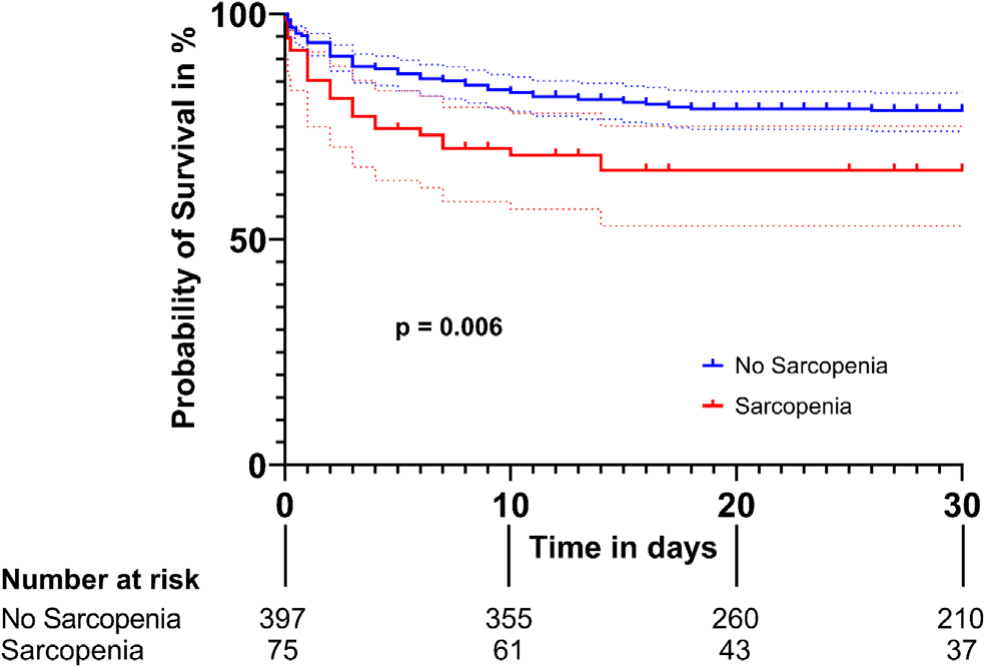
Kaplan–Meier survival curves of patients with and without sarcopenia for all‐cause 30‐day mortality free survival (log‐rank *P* = 0.006).

Regarding 30‐day mortality, significant differences were observed in patients with sarcopenia, visceral obesity and sarcopenic obesity (*Table* *1*). In the Cox proportional hazard model age, ASA classification ≥ III, BMI, sarcopenia, visceral obesity, sarcopenic obesity, ISS and GCS ≤ 8 points were statistically significant variables in univariable analysis (*Table* *2* *a*). In multivariable analysis, significant variables were ASA classification ≥III, BMI, sarcopenia, ISS and GCS ≤ 8 points, while age, visceral obesity and sarcopenic obesity were not statistically significant associated with 30‐day mortality (*Table* *2* *b*). The significance level of BMI changed from statistically significant to statistically not significant after FDR adjustment. The Kaplan–Meier survival curves of patients with and without sarcopenia confirmed a statistically significant difference (*Figure* *2*).

**Table 2:** Cox proportional hazard model of associations with all‐cause 30‐day mortality

Predictor	Coefficients	95% CI	*SE*	*z*	HR	95% CI	*P* value	FDR
(a) Univariable associations								
Male sex	0.01	−0.44	0.45	0.23	1.01	0.64–1.57	0.981	0.981
Age	0.02	0.01–0.03	0.01	4.06	1.02	1.01–1.03	<0.001	<0.001
ASA ≥ III	0.94	0.55–1.34	0.2	4.67	2.56	1.73–3.81	<0.001	<0.001
BMI	0.05	0–0.1	0.03	2.01	1.05	1–1.11	0.044	0.044
ISS	0.06	0.05–0.07	0.01	11.13	1.06	1.05–1.08	<0.001	<0.001
GCS ≤ 8 points	1.98	1.25–2.7	0.37	5.37	7.21	3.51–14.84	<0.001	<0.001
Sarcopenia	0.61	0.16–1.06	0.23	2.67	1.85	1.18–2.89	0.008	0.01
Visceral obesity	0.67	0.27–1.06	0.2	3.32	1.95	1.31–2.89	0.001	0.002
Sarcopenic obesity	0.99	0.4–1.57	0.3	3.32	2.68	1.5–4.8	0.001	0.002
(b) Multivariable associations								
Age	0.01	0–0.03	0.01	1.33	1.01	1–1.03	0.182	0.242
ASA ≥ III	0.88	0.32–1.45	0.29	3.05	2.42	1.37–4.27	0.002	0.005
BMI	0.07	0–0.14	0.03	2.08	1.07	1–1.15	0.038	0.061
ISS	0.07	0.05–0.08	0.01	10.27	1.07	1.06–1.08	<0.001	<0.001
GCS ≤ 8 points	1.28	0.53–2.04	0.38	3.34	3.61	1.7–7.69	0.001	0.004
Sarcopenia	1.05	0.32–1.77	0.37	2.84	2.84	1.38–5.85	0.004	0.008
Visceral obesity	0.03	−0.54‐0.59	0.29	0.09	1.03	0.58–1.81	0.927	0.927
Sarcopenic obesity	−0.47	−1.39‐0.45	0.47	1	0.62	0.25–1.57	0.318	0.363

*Note*: Sarcopenia defined by Prado et al.^14^; visceral obesity defined by Baggerman et al.^22^; sarcopenic obesity defined using definitions of sarcopenia and visceral obesity.^14^, ^22^

Abbreviations: ASA, American Society of Anesthesiologists classification; BMI, body mass index; CI, confidence interval; FDR, false discovery rate; GCS, Glasgow Coma Scale; HR, hazard ratio; ISS, injury severity score; *SE*, standard error.

Direct comparison of patients with and without sarcopenia to detect possible confounding factors revealed significantly higher proportions of male sex, lower median BMI, higher proportions of GCS ≤ 8 points and higher all‐cause 24‐h and 30‐day mortality rates in patients with sarcopenia (*Table* *3*). After FDR adjustment, GCS ≤ 8 points were not statistically significant anymore. Age, ASA classification ≥ III, ISS, ICU LOS and mechanical ventilation duration were comparable in both groups.

**Table 3:** Comparison of patients with and without sarcopenia

Parameter	All patients (*n* = 472)	No sarcopenia (*n* = 397)	Sarcopenia (*n* = 75)	*P* value	FDR
Male, *n* (%)	354 (75)	285 (71.8)	69 (92)	<0.001	<0.001
Age, years; median (IQR)	49 (31; 63.25)	49 (32; 63)	44 (26; 67)	0.444	0.559
ASA ≥ III; *n* (%)	102 (21.6)	84 (21.1)	18 (24)	0.583	0.676
BMI, kg/m^2^; median (IQR)	25.2 (23.4; 27.7)	25.7 (23.5; 27.8)	24.2 (22.9; 24.55)	<0.001	<0.001
Sarcopenia; *n* (%)	75 (15.9)		75 (100)		
Sarcopenia Q1; *n* (%)	115 (24.4)	43 (9.1)	72 (96)	<0.001	<0.001
Visceral obesity; *n* (%)	213 (45.13)	185 (39.2)	28 (37.3)	0.139	0.294
Visceral obesity Q2; *n* (%)	236 (50)	205 (43.4)	31 (6.6)	0.102	0.24
Sarcopenic obesity; *n* (%)	28 (5.9)		28 (37.3)		
Sarcopenic obesity Q1_Q2; *n* (%)	51 (10.8)	23 (5.8)	28 (37.3)	<0.001	<,001
SMM, cm^2^; median (IQR)	180.1 (153.45; 207.15)	189 (161.5; 212.8)	156.4 (140.25; 167.2)	<0.001	<0.001
SMI, cm^2^/m^2^; median (IQR)	57.55 (50.68; 64.4)	59 (53.9; 65.9)	47.9 (44.05; 50.7)	<0.001	<0.001
TAT, cm^2^; median (IQR)	238.25 (143.03; 360.1)	250.1 (146.2; 382.7)	194.3 (114.85; 287.1)	0.001	0.004
SAT, cm^2^; median (IQR)	127.3 (83.15; 181.5)	132.6 (87.5; 188.3)	100.4 (62.05; 139.9)	<0.001	<0.001
VAT, cm^2^; median (IQR)	97.65 (42.95; 179.43)	98.1 (44.1; 190.7	90 (29.1; 140.4)	0.046	0.111
VATI, cm^2^/m^2^; median (IQR)	32.04 (13.76; 57.77)	32.14 (14.17; 61.78)	27.1 (9.65; 42.57)	<0.001	<0.001
ISS; median (IQR)	26 (20; 41)	26 (20; 38)	29 (20.5; 42)	0.193	0.349
AIS head; median (IQR)	3 (0; 5)	3 (0; 5)	4 (0.5; 5)	0.1	0.203
AIS face; median (IQR)	0 (0; 1)	0 (0; 1)	0 (0; 0.5)	0.421	0.554
AIS chest; median (IQR)	3 (0; 4)	3 (0; 4)	3 (2; 4)	0.026	0.068
AIS abdomen, lumbar spine; median (IQR)	0 (0; 2)	0 (0; 2)	0 (0; 2)	0.906	0.906
AIS extremity, pelvis; median (IQR)	2 (0; 3)	2 (0; 3)	1 (0; 3)	0.276	0.444
AIS external; median (IQR)	0 (0; 0)	0 (0; 0)	0 (0; 0)	0.855	0.885
GCS ≤ 8 points; *n* (%)	304 (64.4)	247 (62.2)	57 (76)	0.022	0.063
ICU LOS, days; median (IQR)	8 (2; 22)	8 (3; 21)	7 (2; 24.5)	0.504	0.609
Mechanical ventilation, days; median (IQR)	3 (0.5; 14)	3 (0.5; 13)	4 (0.75; 14.5)	0.671	0.720
24‐h mortality; *n* (%)	36 (7.6)	25 (6.3)	11 (14.6)	0.012	0.038
30‐day mortality; *n* (%)	104 (22)	79 (19.9)	25 (33.3)	0.01	0.036

*Note*: Sarcopenia defined by Prado et al.^14^; visceral obesity defined by Baggerman et al.^22^; sarcopenic obesity defined using definitions of sarcopenia and visceral obesity.^14^, ^22^ Sarcopenia Q1, first quartile of sex‐adjusted SMI of the study cohort associated with 30‐day mortality; visceral obesity Q2, second quartile of sex‐adjusted VATI of the study cohort associated with 30‐day mortality; sarcopenic obesity Q1_Q2, sarcopenic obesity defined by cohort‐specific sarcopenia and visceral obesity definitions.

Abbreviations: AIS, abbreviated injury severity of body region; ASA, American Society of Anesthesiologists classification; BMI, body mass index; FDR, false discovery rate; GCS, Glasgow Coma Scale before tracheal intubation; ICU LOS, intensive care unit length of stay; IQR, interquartile range; ISS, injury severity score; SAT, subcutaneous adipose tissue; SMI, skeletal muscle index; SMM, skeletal muscle mass; TAT, total adipose tissue; VAT, visceral adipose tissue; VATI, visceral adipose tissue index.

A comparison of male and female patients revealed significantly lower proportions of female patients with sarcopenia and sarcopenic obesity, as well as lower BMI values than male patients (*Table* *4*).

**Table 4:** Comparison of male and female patients

Parameter	All patients (*n* = 472)	Males (*n* = 354)	Females (*n* = 118)	*P* value	FDR
Age, years; median (IQR)	49 (31; 63.25)	47.5 (30.25; 62.75)	52 (32; 70)	0.101	0.227
ASA ≥ III; *n* (%)	102 (21.6)	69 (19.5)	33 (27.9)	0.053	0.130
BMI, kg/m^2^; median (IQR)	25.2 (23.4; 27.7)	25.5 (23.7; 27.7)	24.4 (21.5; 27.7)	0.007	0.021
Sarcopenia; *n* (%)	75 (15.9)	69 (19.5)	6 (5.1)	<0.001	0.002
Sarcopenia Q1; *n* (%)	115 (24.4)	86 (24.3)	29 (24.5)	0.951	1
Visceral obesity; *n* (%)	213 (45.1)	162 (45.7)	51 (43.2)	0.631	0.903
Visceral obesity Q2; *n* (%)	236 (50)	177 (50)	59 (50)	1	1
Sarcopenic obesity; *n* (%)	28 (5.9)	27 (7.6)	1 (0.8)	0.007	0.021
Sarcopenic obesity Q1_Q2; *n* (%)	51 (10.8)	37 (10.4)	14 (11.9)	0.669	0.861
SMM, cm^2^; median (IQR)	180.1 (153.45; 207.15)	192.8 (172.7; 215.6)	139.3 (124.1; 152.4)	<0.001	<0.001
SMI, cm^2^/m^2^; median (IQR)	57.55 (50.68; 64.4)	59.5 (53.85; 66)	49.75 (44.73; 54.75)	<0.001	<0.001
TAT, cm^2^; median (IQR)	238.25 (143.03; 360.1)	248.15 (142.9; 362.7)	198.2 (143.6; 354.67)	0.503	0.848
SAT, cm^2^; median (IQR)	127.3 (83.15; 181.5)	119.85 (75.4; 166.9)	146.15 (106.2; 233.4)	<0.001	<0.001
VAT, cm^2^; median (IQR)	97.65 (42.95; 179.43)	116.65 (50.2; 196.75)	54.6 (56.6; 111.4)	<0.001	<0.001
VATI, cm^2^/m^2^; median (IQR)	32 (13.76; 57.8)	36.4 (15.7; 62.3)	18.9 (9.5; 40.1)	<0.001	<0.001
ISS; median (IQR)	26 (20; 41)	25 (29; 40.25)	29 (20; 41.75)	0.357	0.688
AIS head; median (IQR)	3 (0; 5)	3 (0; 5)	3 (1; 4)	0.913	1
AIS face; median (IQR)	0 (0; 1)	0 (0; 1.75)	0 (0; 1)	0.456	0.82
AIS chest; median (IQR)	3 (0; 4)	3 (0; 4)	3 (0; 4)	0.642	0.903
AIS abdomen, lumbar spine; median (IQR)	0 (0; 2)	0 (0; 2)	0 (0; 3)	0.012	0.032
AIS extremity, pelvis; median (IQR)	2 (0; 3)	1 (0; 3)	3 (0; 4)	0.003	0.012
AIS external; median (IQR)	0 (0; 0)	0 (0; 0)	0 (0; 0)	0.564	0.895
GCS ≤ 8 points; *n* (%)	304 (64.4)	227 (64.1)	77 (56.3)	0.824	1
ICU LOS, days; median (IQR)	8 (3; 22)	8 (3; 21.75)	3 (8.5; 21.5)	0.731	0.939
Mechanical ventilation, days; median (IQR)	3 (0.5; 14)	3 (0.5; 15)	3 (0.5; 10)	0.218	0.452
24‐h mortality; *n* (%)	36 (7.6)	27 (7.6)	9 (7.6)	1	1
30‐day mortality; *n* (%)	104 (22)	78 (22)	26 (22)	1	1

*Note*: Sarcopenia defined by Prado et al.^14^; visceral obesity defined by Baggerman et al.^22^; sarcopenic obesity defined using definitions of sarcopenia and visceral obesity.^14^, ^22^ Sarcopenia Q1, first quartile of sex‐adjusted SMI of the study cohort associated with 30‐day mortality; visceral obesity Q2, second quartile of sex‐adjusted VATI of the study cohort associated with 30‐day mortality; sarcopenic obesity Q1_Q2, sarcopenic obesity defined by cohort‐specific sarcopenia and visceral obesity definitions.

Abbreviations: AIS, abbreviated injury scale of body region; ASA, American Society of Anesthesiologists classification; BMI, body mass index; FDR, false discovery rate; GCS, Glasgow Coma Scale; ICU LOS, intensive care unit length of stay; IQR, interquartile range; ISS TAT, total adipose tissue; ISS, injury severity score; SAT, subcutaneous adipose tissue; SMI, skeletal muscle index; SMM, skeletal muscle mass; VAT, visceral adipose tissue; VATI, visceral adipose tissue index.

In the subgroup of survivors, associations with ICU LOS and mechanical ventilation duration were analysed. Univariable linear regression analysis identified age, ISS and GCS ≤ 8 points as significant associations with ICU LOS, while ASA classification ≥ III and visceral obesity did not reach significance level when FDR was applied (*Table* *5* *a*). No significant associations were observed for BMI, sarcopenia and sarcopenic obesity. Multivariable analysis including significant predictors from univariable analyses confirmed age, ISS and GCS ≤ 8 points as independent associations (*Table* *5*).

**Table 5:** Linear regression analysis of associations with ICU LOS in 368 survivors

Predictor	*B*	*β*	*SE*	*t*	95% CI for *B*	*P* value	FDR
(a) Univariable associations							
Age	0.18	0.21	0.04	4.09	0.09–0.27	<0.001	<0.001
ASA ≥ III	4.51	0.11	2.23	2.03	0.11–8.92	0.044	0.068
BMI	0.08	0.02	0.24	0.35	−0.39‐0.55	0.724	0.724
ISS	0.67	0.52	0.06	11.75	0.56–0.78	<0.001	<0.001
GCS ≤ 8 points	−9.45	−0.29	1.62	−5.84	−12.65 to −6.25	<0.001	<0.001
Sarcopenia	2.92	0.06	2.44	1.19	−1.91 to 7.75	0.233	0.266
Visceral obesity	3.39	0.1	1.7	2	0.03–6.74	0.047	0.068
Sarcopenic obesity	8.55	0.1	4.36	1.96	−0.08 to 17.17	0.051	0.068
(b) Multivariable associations							
Age	0.21	0.25	0.04	5.83	0.14–0.29	<0.001	<0.001
ISS	0.67	0.52	0.06	11.99	0.56–0.77	<0.001	<0.001
GCS ≤ 8 points	4.06	0.13	1.42	2.85	1.25–6.88	0.005	0.005

*Note*: Sarcopenia defined by Prado et al.^14^; visceral obesity defined by Baggerman et al.^22^; sarcopenic obesity defined using definitions of sarcopenia and visceral obesity.^14^, ^22^

Abbreviations: ASA, American Society of Anesthesiologists classification; *B*, unstandardized coefficient; BMI, body mass index; CI, confidence interval; FDR, false discovery rate; GCS, Glasgow Coma Scale; ICU LOS, intensive care unit length of stay; ISS, injury severity score; *SE*, standard error; *β*, standardized coefficient.

Statistically significant univariable associations with mechanical ventilation duration were age, ASA classification ≥ III, ISS, GCS ≤ 8 points, visceral obesity and sarcopenic obesity while BMI and sarcopenia were not (*Table* *6* *a*). Multivariable analysis including significant predictors from univariable analyses confirmed age, ISS and GCS ≤ 8 points as independent predictors whereas no significant associations were found for the ASA classification ≥ III, visceral obesity and sarcopenic obesity (*Table* *6* *b*). All multivariable associations remained significant after FDR adjustment.

**Table 6:** Linear regression analysis of associations with mechanical ventilation duration in 368 survivors

Predictor	*B*	*β*	*SE*	*t*	95% CI for *B*	*P* value	FDR
(a) Univariable associations							
Age	0.11	0.2	0.03	3.96	0.06–0.17	<0.001	<0.001
ASA ≥ III	3.78	0.13	1.46	2.59	0.89–6.67	0.01	0.016
BMI	0.16	0.06	0.15	1.05	−0.14 to 0.47	0.294	0.336
ISS	0.41	0.49	0.04	10.63	0.33–0.48	<0.001	<0.001
GCS ≤ 8 points	8.51	0.41	1.01	8.44	6.51–10.5	<0.001	<0.001
Sarcopenia	1.2	0.04	1.6	0.75	−1.97 to 4.37	0.454	0.454
Visceral obesity	3.43	0.15	1.19	2.88	1.08–5.78	0.004	0.008
Sarcopenic obesity	5.47	0.11	2.53	2.16	0.46–10.48	0.031	0.041
(b) Multivariable associations							
Age	0.14	0.23	0.04	3.6	0.06–0.21	<0.001	<0.001
ASA ≥ III	−0.32	−0.01	1.68	−0.19	−3.63 to 3	0.851	0.924
ISS	0.39	0.43	0.04	9.91	0.31–0.47	<0.001	<0.001
GCS ≤ 8 points	6.03	0.26	1	6	4.04–8.01	<0.001	<0.001
Visceral obesity	0.68	0.03	1.18	0.57	−1.65 to 3.01	0.566	0.849
Sarcopenic obesity	0.24	0	2.56	0.1	−4.81 to 5.3	0.924	0.924

*Note*: Sarcopenia defined by Prado et al.^14^; visceral obesity defined by Baggerman et al.^22^; sarcopenic obesity defined using definitions of sarcopenia and visceral obesity.^14^, ^22^

Abbreviations: ASA, American Society of Anesthesiologists classification; *B*, unstandardized coefficient; BMI, body mass index; CI, confidence interval; FDR, false discovery rate; GCS, Glasgow Coma Scale; ISS, injury severity score; *SE*, standard error; *β*, standardized coefficient.

A cohort‐specific definition of sarcopenia identified the first quartile of sex‐adjusted SMI as the most significant univariable association with 30‐day mortality (males <53.85 cm^2^/m^2^ and females <44.73 cm^2^/m^2^) (*Tables* *1* and *S1*). After multivariable Cox regression analysis, the statistically significant association of the cohort‐specific sarcopenia definition with 30‐day mortality was not confirmed. The cohort‐specific sarcopenia definition was not found to be associated with ICU LOS or mechanical ventilation duration (*Table* *S2*).

The second quartile of sex‐adjusted VATI demonstrated the strongest association with 30‐day mortality, thus defining visceral obesity as >36.38 cm^2^/m^2^ for males and >18.87 cm^2^/m^2^ for females. (*Tables* *1* and *S1*). This cohort‐specific visceral obesity definition was also found to be significantly associated with ICU LOS and mechanical ventilation duration in univariable linear regression analysis. After adjustment for FDR, the association with ICU LOS was not significant. In multivariable analysis, neither the association with 30‐day mortality nor the association with mechanical ventilation duration was confirmed (*Tables* *S1* and *S2*).

Cohort‐specific sarcopenic obesity, defined as the combination of sarcopenia and visceral obesity according to the best matching quartiles of the cohort, was found to be significantly associated with 30‐day mortality, ICU LOS and mechanical ventilation time in univariable analyses (*Table* *S1*). The results of multivariable analyses did not confirm these associations (*Table* *S2*).

## Discussion

The present results suggest that sarcopenia is an independent predictor of 30‐day mortality in a cohort of severely injured patients requiring emergency tracheal intubation and mechanical ventilation. Available studies on CT‐defined body composition parameters in trauma patients report divergent associations with mortality, whereas low sample sizes, different definitions, inconsistent injury severity and missing data are limiting factors throughout the literature.^17^, ^18^, ^19^, ^25^, ^26^, ^27^, ^28^, ^29^, ^30^, ^31^, ^32^, ^33^ Although some studies found statistically significant associations of body composition parameters with overall mortality, particularly in elderly and mechanically ventilated patients,^19^, ^25^, ^26^, ^27^, ^28^, ^29^, ^30^ other studies could only indicate prognostic effects for non‐mortality complications (i.e., ICU LOS and mechanical ventilation duration)^19^, ^32^, ^33^, ^34^, ^35^ or did not find statistically significant associations.^34^ In this study, previously defined body composition parameters were not statistically significant associated with ICU LOS while a statistically significant association of visceral obesity and sarcopenic obesity with mechanical ventilation duration was found in univariable analysis, but not after adjustment for multiple predictors. Similarly, newly defined thresholds of the study cohort using the first quartile of sex‐adjusted SMI for sarcopenia, the second quartile of sex‐adjusted VATI for visceral obesity and cohort‐specific sarcopenic obesity showed statistically significant associations in univariable analysis but not in multivariable analysis of 30‐day mortality, ICU LOS and mechanical ventilation duration. The failure to confirm the statistically significant association of sarcopenia using the new definition of the first quartile compared with the definition by Prado et al. indicates that the results of this study should be interpreted with caution and that further prospective studies with larger sample sizes and more balanced distribution of male and female patients are necessary.

In a study of an Asian population of 939 trauma patients, sarcopenia (defined as an extremely low psoas muscle index) was identified as an independent risk factor for a longer ICU LOS (β coefficient = 3.881, *P* = 0.011).^32^ However, the sarcopenia assessment with every muscle included at lumbar level L3 is the preferred method in the literature, whereas the psoas muscle measurement has some limitations and is not as standardized.

A recent study from Aachen, Germany, found SMI at L3 as significant prognostic factor of overall mortality, along with highly significant predictors age and ISS.^28^ Interestingly, the diagnostic capabilities of SMI at thoracic vertebra Th4 were comparable with measurements at lumbar level L3. Furthermore, SMI was associated with posttraumatic complications and systemic infections, whereas SAT index was associated with the duration of mechanical ventilation.

In contrast to the previously mentioned study and the results presented here, another complex analysis of 297 trauma patients from Munich, Germany, revealed no significant effects of abdominal fat and muscle tissue parameters for ICU LOS, mechanical ventilation duration or neurological outcome although acute emergency care and early immune response were influenced by obesity.^33^

These contradictory findings might be related to the different injury severities and mortality rates of included patients and to the relatively low sample sizes of the study cohorts, which implicates the need for prospective studies involving higher numbers of patients.

Studies, in which other muscle parameters than lumbar level L3 were evaluated for body composition analysis, the previously mentioned thoracic vertebra level Th4^28^ and masseter muscle,^35^ revealed promising results. These approaches might be preferred for patients undergoing only CT imaging of the thorax and neck.

A recent evaluation of a deep learning method for automatic segmentation of CT slices at the L3 lumbar vertebra level in polytrauma patients revealed promising results regarding time efficiency, consistency and accuracy.^36^ The deep learning neural network, containing the data of a multi‐centre sample of 3413 abdominal cancer surgery patients to automatically calculate muscle and adipose tissue, was applied on 536 polytrauma patients as an independent test cohort. Compared with a human expert operator, the deep learning results had excellent agreement for all body composition indices. Further developments of automation using deep learning‐based diagnostic approaches might help in implementing complex quantitative body composition assessments into clinical routines in the near future.

The present analysis has several limitations. First, retrospective studies are prone to possible inherent bias. Second, the patient sample was based on a single trauma centre, which may cause selection bias. Third, only patients requiring tracheal intubation and who underwent whole‐body CT diagnostics were included in this analysis. Patients without emergency tracheal intubation and mechanical ventilation, and those who did not receive initial whole‐body CT diagnostics due to direct transfer from the resuscitation room to the operating room, undergoing only head and/or chest CT, or who died in the resuscitation room may have presented with other injury predictors and had different outcomes. Fourth, surgical procedures and complications during ICU LOS were not included in this analysis. Fifth, although the body composition analysis was performed semiquantitatively as can be considered the current scientific standard, we cannot exclude investigator‐related bias. Sixth, although the sarcopenia definition by Prado et al. is a commonly accepted definition in the literature, a significantly lower proportion of female patients were classified as sarcopenic in this study cohort. This may be the result of the relatively low sample size. The use of a novel threshold based on the most significantly associated sex‐adjusted quartile with 30‐day mortality may not be the optimal approach for defining sarcopenia in general, due to possible selection bias. Moreover, the main objective of this study was not to generate new threshold values but to validate a highly selected cohort of severely injured and mechanically ventilated trauma patients against established and accepted body composition parameter definitions. Seventh, the frequency of visceral obesity in the present cohort was high but comparable with the published literature. For example, recently conducted meta‐analyses regarding the effect of visceral obesity on surgical complications reported similar frequencies of visceral obesity.^37^, ^38^ Furthermore, the employed threshold by the definition of Baggerman et al. employs an index and is therefore more comparable with the sarcopenia definition rather than other threshold values (e.g., >100 cm^2^).^21^, ^22^ Similarly, the identified frequency of sarcopenic patients is in line with the published literature.^39^ Eighth, the data required to calculate outcome probability were not available due to the retrospective nature of the study. Consequently, APACHE II scores and other valuable scores (e.g., RISC II score) could not be provided.

## Conclusions

Sarcopenia measured via initial whole‐body CT was associated with 30‐day mortality in severely injured trauma patients requiring emergency tracheal intubation and mechanical ventilation, suggesting that routine measurement of body composition parameters might help to identify patients at risk. In the subgroup of survivors, associations of body composition parameters with ICU LOS and mechanical ventilation duration were not observed.

## Conflicts of interest

The authors declare that they have no competing interests.

## Funding

There was no funding for this study.

## Consent

Consent to participate was not applicable due to the retrospective nature of the study. The need for informed consent was waived by the Institutional Review Board of the study centre.

## Supporting information


**Table S1** Univariable determination of cohort‐specific body composition parameters

**Table S2** Multivariable validation of cohort‐specific of body composition parameters
