# Supplementary material for: Body composition parameters in initial CT imaging of mechanically ventilated trauma patients: Single‐centre observational study
Source: J Cachexia Sarcopenia Muscle. 2024 Aug 26;15(6):2437–46. doi: 10.1002/jcsm.13578 (PMC11634470; doi:10.1002/jcsm.13578)
Supplement: Supplementary file 1 — Table S1 Univariable determination of cohort‐specific body composition parameters [file JCSM-15-2437-s002.docx]

**Table S1 Univariable determination of cohort-specific body composition parameters**

1. **Univariable Cox regression associations of body composition parameters with 30-day mortality in 472 mechanically ventilated trauma patients**

| Predictor | Coefficients | 95% CI | SE | z | HR | 95% CI | p-value | FDR |
| --- | --- | --- | --- | --- | --- | --- | --- | --- |
| Sarcopenia according to Prado | 0.61 | 0.16-1.06 | 0.23 | 2.67 | 1.85 | 1.18-2.89 | **0.008** | **0.015** |
| Sarcopenia according to quartile 1 | 0.51 | 0.1-0.92 | 0.21 | 2.46 | 1.67 | 1.11-2.5 | **0.014** | **0.019** |
| Sarcopenia according quartile 2 | 0.4 | 0.01-0.79 | 0.2 | 1.99 | 1.49 | 1.01-2.2 | **0.046** | **0.051** |
| Sarcopenia according to quartile 3 | -0.18 | -0.66-0.29 | 0.24 | 0.75 | 0.83 | 0.52-1.34 | 0.451 | 0.451 |
| Visceral obesity according to Baggerman | 0.67 | 0.27-1.06 | 0.2 | 3.32 | 1.95 | 1.31-2.89 | **0.001** | **0.002** |
| Visceral obestiy according to quartile 1 | 0.66 | 0.13-1.19 | 0.27 | 2.43 | 1.93 | 1.13-3.29 | **0.015** | **0.019** |
| Visceral obesity according to quartile 2 | 0.53 | 0.13-0.93 | 0.2 | 2.62 | 1.7 | 1.14-2.53 | **0.009** | **0.015** |
| Visceral obesity according to quartile 3 | 0.44 | 0.04-0.85 | 0.21 | 2.13 | 1.56 | 1.04-2.34 | **0.033** | **0.039** |
| Sarcopenic obesity according to Prado and Baggerman | 0.99 | 0.4-1.57 | 0.3 | 3.32 | 2.68 | 1.5-4.8 | **0.001** | **0.002** |
| Sarcopenic obesity according to Prado and visceral obesity quartile 2 | 0.98 | 0.43-1.53 | 0.28 | 3.51 | 2.66 | 1.54-4.6 | **<0.001** | **<0.001** |
| Sarcopenic obesity according to quartile 1 and Baggerman | 0.98 | 0.5-1.46 | 0.24 | 4.01 | 2.67 | 1.65-4.3 | **<0.001** | **<0.001** |
| Sarcopenic obesity according to quartile 1 and quartile 2 | 0.87 | 0.4-1.34 | 0.24 | 3.63 | 2.39 | 1.49-3.83 | **<0.001** | **<0.001** |

1. **Univariable linear regression analysis of associations of body composition parameters with ICU LOS in 368 survivors**

| Predictor | B | β | SE | t | 95% CI for B | p-value | FDR |
| --- | --- | --- | --- | --- | --- | --- | --- |
| Sarcopenia according to Prado | 2.92 | 0.06 | 2.44 | 1.19 | -1.91-7.75 | 0.233 | 0.279 |
| Sarcopenia according to quartile 1 | 3.51 | 0.09 | 2.02 | 1.73 | -0.5-7.51 | 0.084 | 0.144 |
| Sarcopenia according quartile 2 | 1.24 | 0.04 | 1.68 | 0.74 | -2.08-4.56 | 0.461 | 0.502 |
| Sarcopenia according to quartile 3 | 2.42 | 0.07 | 1.92 | 1.26 | -1.37-6.21 | 0.208 | 0.277 |
| Visceral obesity according to Baggerman | 3.39 | 0.1 | 1.7 | 2 | 0.03-6.74 | **0.047** | 0.102 |
| Visceral obestiy according to quartile 1 | 0.95 | 0.03 | 1.87 | 0.51 | -2.74-4.64 | 0.612 | 0.612 |
| Visceral obesity according to quartile 2 | 3.68 | 0.11 | 1.67 | 2.2 | 0.38-6.98 | **0.028** | 0.084 |
| Visceral obesity according to quartile 3 | 3.03 | 0.08 | 1.99 | 1.52 | -0.91-6.97 | 0.129 | 0.193 |
| Sarcopenic obesity according to Prado and Baggerman | 8.55 | 0.1 | 4.36 | 1.96 | -0.08-17.17 | 0.051 | 0.102 |
| Sarcopenic obesity according to Prado and visceral obesity quartile 2 | 12.69 | 0.17 | 3.94 | 3.22 | 4.9-20.48 | **0.001** | **0.008** |
| Sarcopenic obesity according to quartile 1 and Baggerman | 10.53 | 0.16 | 3.35 | 3.14 | 3.91-17.16 | **0.002** | **0.008** |
| Sarcopenic obesity according to quartile 1 and quartile 2 | 9.45 | 0.16 | 3.07 | 3.08 | 3.37-15.52 | **0.002** | **0.008** |

1. **Univariable linear regression analysis of associations of body composition parameters with mechanical ventilation duration in 368 survivors**

| Predictor | B | β | SE | t | 95% CI for B | p-value | FDR |
| --- | --- | --- | --- | --- | --- | --- | --- |
| Sarcopenia according to Prado | 1.2 | 0.04 | 1.6 | 0.75 | -1.97-4.37 | 0.454 | 0.504 |
| Sarcopenia according to quartile 1 | 2.59 | 0.09 | 1.43 | 1.82 | -0.23-5.41 | 0.07 | 0.116 |
| Sarcopenia according quartile 2 | 1.76 | 0.08 | 1.18 | 1.5 | -0.57-4.1 | 0.135 | 0.192 |
| Sarcopenia according to quartile 3 | 1.9 | 0.07 | 1.35 | 1.41 | -0.77-4.57 | 0.16 | 0.2 |
| Visceral obesity according to Baggerman | 3.43 | 0.15 | 1.19 | 2.88 | 1.08-5.78 | **0.004** | **0.012** |
| Visceral obesity according to quartile 1 | 0.43 | 0.02 | 1.32 | 0.32 | -2.18-3.03 | 0.747 | 0.747 |
| Visceral obesity according to quartile 2 | 3.29 | 0.15 | 1.17 | 2.81 | 0.97-5.61 | **0.005** | **0.012** |
| Visceral obesity according to quartile 3 | 3.55 | 0.13 | 1.4 | 2.54 | 0.79-6.31 | **0.011** | **0.022** |
| Sarcopenic obesity according to Prado and Baggerman | 5.47 | 0.11 | 2.53 | 2.16 | 0.46-10.48 | **0.031** | 0.053 |
| Sarcopenic obesity according to Prado and visceral obesity quartile 2 | 7.79 | 0.14 | 2.79 | 2.8 | 2.28-13.3 | **0.005** | **0.012** |
| Sarcopenic obesity according to quartile 1 and Baggerman | 7.08 | 0.15 | 2.36 | 2.99 | 2.4-11.76 | **0.003** | **0.012** |
| Sarcopenic obesity according to quartile 1 and quartile 2 | 6.92 | 0.16 | 2.16 | 3.2 | 2.64-11.2 | **0.002** | **0.012** |

SE, standard error; CI, confidence interval; HR, hazard ratio; B, unstandardized coefficient; β, standardized coefficient; FDR, false discovery rate; ICU LOS, intensive care unit length of stay; Quartiles 1-3, sex-adjusted quartiles of univariable associations with 30-day mortality of the study cohort; Prado, Prado et al. [14]; Baggerman, Baggerman et al. [22].
