# Supplementary material for: Body composition parameters in initial CT imaging of mechanically ventilated trauma patients: Single‐centre observational study
Source: J Cachexia Sarcopenia Muscle. 2024 Aug 26;15(6):2437–46. doi: 10.1002/jcsm.13578 (PMC11634470; doi:10.1002/jcsm.13578)
Supplement: Supplementary file 2 — Table S2 Multivariable validation of cohort‐specific of body composition parameters [file JCSM-15-2437-s001.docx]

**Table S2 Multivariable validation of cohort-specific body composition parameters**

1. **Multivariable Cox regression analysis of associations of body composition parameters with 30-day mortality in 472 mechanically ventilated trauma patients**

| Predictor | Coefficients | 95% CI | SE | z | HR | 95% CI | p-value | FDR |
| --- | --- | --- | --- | --- | --- | --- | --- | --- |
| Age | 0.01 | -0.01-0.02 | 0.01 | 0.94 | 1.01 | 0.99-1.02 | 0.346 | 0.518 |
| ASA ≥ III | 0.87 | 0.3-1.43 | 0.29 | 2.99 | 2.38 | 1.35-4.2 | **0.003** | **0.008** |
| BMI | 0.06 | -0.01-0.12 | 0.03 | 1.66 | 1.06 | 0.99-1.13 | 0.097 | 0.194 |
| ISS | 0.06 | 0.05-0.08 | 0.01 | 10.18 | 1.07 | 1.05-1.08 | **<0.001** | **<0.001** |
| GCS ≤ 8 points | -1.26 | -2.01—0.5 | 0.38 | 3.27 | 0.28 | 0.13-0.6 | **0.001** | **0.004** |
| Sarcopenia according to quartile 1 | -0.26 | -0.94-0.42 | 0.35 | 0.75 | 0.77 | 0.39-1.52 | 0.454 | 0.518 |
| Visceral obesity according to quartile 2 | 0 | -0.62-0.62 | 0.32 | 0.01 | 1 | 0.54-1.87 | 0.994 | 0.994 |
| Sarcopenic obesity according to quartiles 1 and 2 | 0.34 | -0.4-1.18 | 0.43 | 0.79 | 1.4 | 0.6-3.26 | 0.43 | 0.518 |

1. **Multivariable linear regression analysis of associations of body composition parameters with ICU LOS in 368 survivors**

| Predictor | B | β | SE | t | 95% CI for B | p-value | FDR |
| --- | --- | --- | --- | --- | --- | --- | --- |
| Age | 0.2 | 0.23 | 0.04 | 5.31 | 0.13-0.27 | **<0.001** | **<0.001** |
| ISS | 0.66 | 0.52 | 0.06 | 11.92 | 0.55-0.77 | **<0.001** | **<0.001** |
| GCS ≤ 8 points | 4 | 0.12 | 1.42 | 2.81 | 1.19-6.8 | **0.005** | **0.007** |
| Sarcopenic obesity according quartiles 1 and 2 | 4.9 | 0.08 | 2.63 | 1.87 | -0.29-10.1 | 0.053 | 0.053 |

1. **Multivariable linear regression analysis of associations of body composition parameters with mechanical ventilation duration in 368 survivors**

| Predictor | B | β | SE | t | 95% CI for B | p-value | FDR |
| --- | --- | --- | --- | --- | --- | --- | --- |
| Age | 0.22 | 0.26 | 0.06 | 3.99 | 0.11-0.33 | **<0.001** | **<0.001** |
| ASA ≥ III | -1.57 | -0.04 | 2.37 | -0.66 | -6.26-3.12 | 0.508 | 0.609 |
| ISS | 0.66 | 0.52 | 0.06 | 11.86 | 0.55-0.77 | **<0.001** | **<0.001** |
| GCS ≤ 8 points | 3.99 | 0.12 | 1.42 | 2.81 | 1.18-6.8 | **0.005** | **0.01** |
| Visceral obesity according to quartile 2 | -0.02 | 0 | 1.67 | -0.01 | -3.33-3.29 | 0.992 | 0.992 |
| Sarcopenic obesity according to quartiles 1 and 2 | 4.89 | 0.08 | 2.63 | 1.86 | -0.31-10.09 | 0.064 | 0.096 |

ICU LOS, intensive care unit length of stay; B, unstandardized coefficient; β, standardized coefficient; SE, standard error; CI, confidence interval; FDR, false discovery rate; ASA, American Society of Anesthesiologists classification; BMI, body mass index; ISS, injury severity score, GCS, Glasgow coma scale. Sarcopenia definition according to sex-adjusted first quartile of skeletal muscle index of univariable association with 30-day mortality of the study cohort; Visceral obesity definition according to sex-adjusted second quartile of visceral adipose tissue index of univariable association with 30-day mortality of the study cohort; Sarcopenic obesity definition using sarcopenia and visceral obesity definition of the study cohort.
